# Machine learning optimization of peptides for presentation by class II MHCs

**DOI:** 10.1093/bioinformatics/btab131

**Published:** 2021-03-11

**Authors:** Zheng Dai, Brooke D Huisman, Haoyang Zeng, Brandon Carter, Siddhartha Jain, Michael E Birnbaum, David K Gifford

**Affiliations:** Computer Science and Artificial Intelligence Laboratory, MIT, Cambridge, MA, USA; Department of Computer Science and Electrical Engineering, MIT, Cambridge, MA, USA; Department of Biological Engineering, MIT, Cambridge, MA, USA; Computer Science and Artificial Intelligence Laboratory, MIT, Cambridge, MA, USA; Department of Computer Science and Electrical Engineering, MIT, Cambridge, MA, USA; Computer Science and Artificial Intelligence Laboratory, MIT, Cambridge, MA, USA; Department of Computer Science and Electrical Engineering, MIT, Cambridge, MA, USA; Computer Science and Artificial Intelligence Laboratory, MIT, Cambridge, MA, USA; Department of Computer Science and Electrical Engineering, MIT, Cambridge, MA, USA; Department of Biological Engineering, MIT, Cambridge, MA, USA; Computer Science and Artificial Intelligence Laboratory, MIT, Cambridge, MA, USA; Department of Computer Science and Electrical Engineering, MIT, Cambridge, MA, USA; Department of Biological Engineering, MIT, Cambridge, MA, USA

## Abstract

**Summary:**

T cells play a critical role in cellular immune responses to pathogens and cancer and can be activated and expanded by Major Histocompatibility Complex (MHC)-presented antigens contained in peptide vaccines. We present a machine learning method to optimize the presentation of peptides by class II MHCs by modifying their anchor residues. Our method first learns a model of peptide affinity for a class II MHC using an ensemble of deep residual networks, and then uses the model to propose anchor residue changes to improve peptide affinity. We use a high throughput yeast display assay to show that anchor residue optimization improves peptide binding.

**Supplementary information:**

Supplementary data are available at *Bioinformatics* online.

## 1 Introduction

Machine learning holds great promise for improving therapeutic molecules, and here, we show how it can be applied to enhance the display of peptides that invoke cellular immune responses to pathogens and cancer. T cells surveille peptides displayed on the cell surface by Major Histocompatibility Complexes (MHCs), or Human Leukocyte Antigens (HLAs) in humans, and T cell-mediated killing is initiated by recognition of a foreign peptide bound to an MHC. Specifically, CD8+ cytotoxic T cells recognize peptides presented by class I MHCs (MHCI), and CD4+ helper T cells recognize peptides presented by class II MHCs (MHCII) (Hennecke and Wiley, 2001). MHCIs have a closed peptide-binding groove and typically present peptides of 8–11 amino acids; MHCIIs have an open binding groove and typically present longer peptides, with a 9 amino acid core binding within the groove and the ends protruding from the groove. Prediction algorithms have been utilized to predict peptide-MHC binding. Recent strides in algorithmic performance have been enabled by advances in computational methods (Chen *et al.*, 2019; O’Donnell *et al.*, 2020; Racle *et al.*, 2019; Reynisson *et al.*, 2020; Zeng and Gifford, 2019) and the development of new methodologies for generating training data, such as mono-allelic mass spectrometry (Abelin *et al.*, 2019, 2017; Sarkizova *et al.*, 2020) and yeast display (Rappazzo *et al.*, 2020). With the help of these tools, peptide vaccines with constituent peptides computationally selected for the ability to be displayed by MHCs have been utilized to amplify T cell responses and proven clinically successful for patients with cancer after eliciting CD8+ and CD4+ T cell responses (Abelin *et al.*, 2017; Hu *et al.*, 2018; Ott *et al.*, 2017).

Engineered peptides with modified residues can further improve the effectiveness of such interventions. It has been observed that peptides with modified peptide anchor residues can improve the tumor cell killing response of the adaptive immune system (van Stipdonk *et al.*, 2009). For peptides presented by MHCI, not all modifications to the antigen sequences improve the recognition of peptides by the immune system, likely due to subtle structural changes that alter the T-cell receptor binding interface (Cole *et al.*, 2010). However, in contrast to MHCIs, MHCIIs have open grooves in which presented peptides are displayed in an extended conformation, resulting in peptides binding in a highly conserved manner. The peptide side chains at positions P1, P4, P6 and P9 are completely buried within binding pockets in the groove and are considered anchor positions (Jones *et al.*, 2006). These four anchor residues are key determinants of peptide-MHC binding affinity. Because of the highly conserved conformation of peptides within the MHCII-binding groove (Jones *et al.*, 2006), changing the identities of the MHCII-binding anchor residues will allow us to alter binding affinity without changing binding conformation or the T cell receptor interface.

Traditional approaches to identifying good peptide modifications is a complicated process (van Stipdonk *et al.*, 2009), necessitating the development of computational approaches. A rule based approach, EpitOptimizer, has been used to design modified peptides for MHCI binding with anchor position changes that resulted in improved adaptive immune system response (Houghton *et al.*, 2007). EpitOptimizer uses a limited sequence context for its suggestions, and each MHCI molecule has a different set of rules. By contrast, PeptX (Knapp *et al.*, 2011) uses a genetic algorithm to determine the peptides most likely to be displayed by a specific MHCI allele, which may provide helpful information for the subsequent design of a vaccine. The performance of PeptX was not experimentally evaluated.

We introduce a model-based approach to optimize peptide-MHCII binding by optimizing the peptide anchor residues of disease-associated peptides. We optimize peptide-MHCII affinity by enumerating all possible changes to the anchor positions of a peptide, then scoring them against an objective function in silico and choosing the best ones. We utilized a yeast display platform for testing our improved peptide sequences for binding to MHCII molecules. This is computationally tractable due to the limited number of anchor positions on a given peptide.

For our objective function, we use predictions from the PUFFIN peptide-MHC binding model (Zeng and Gifford, 2019) trained on peptide-binding data from a MHCII yeast display platform (Rappazzo *et al.*, 2020). PUFFIN uses an ensemble of deep residual networks to quantify its uncertainty about its predictions, while achieving state-of-the-art performance on MHCII-binding prediction tasks (Zeng and Gifford, 2019). We show that our method generates peptide modifications that improve peptide-binding affinity for two MHCIIs.

## 2 Materials and methods

### 2.1 We evaluate the complete anchor substitution landscape with a machine learning model

For a given MHCII, we train a neural network-based machine learning (ML) model (PUFFIN) (Zeng and Gifford, 2019) that takes a nine residue peptide sequence as input, and outputs a measure of the strength of the peptide-MHC interaction. We selected the PUFFIN architecture because it outputs uncertainty estimates, which allows us to compute Bayesian acquisition functions. PUFFIN achieves this by outputting a distribution instead of a value (Zeng and Gifford, 2019), dropout (Gal and Ghahramani, 2016) and ensemble methods (Lakshminarayanan *et al.*, 2017). We leverage the relatively small space of $20^{4}-1$ possible anchor substitutions to evaluate an objective function over each substitution based on the output of the model. We then output the 10 substitutions that score the highest as the proposed optimizations. The use of a neural network-based model along with the complete enumeration of the anchor substitution space allows our optimizations to take more complex interactions between residues into account.

### 2.2 Data were collected using a high throughput peptide display assay that measures enrichment as a surrogate for affinity

**Fig. 1.**
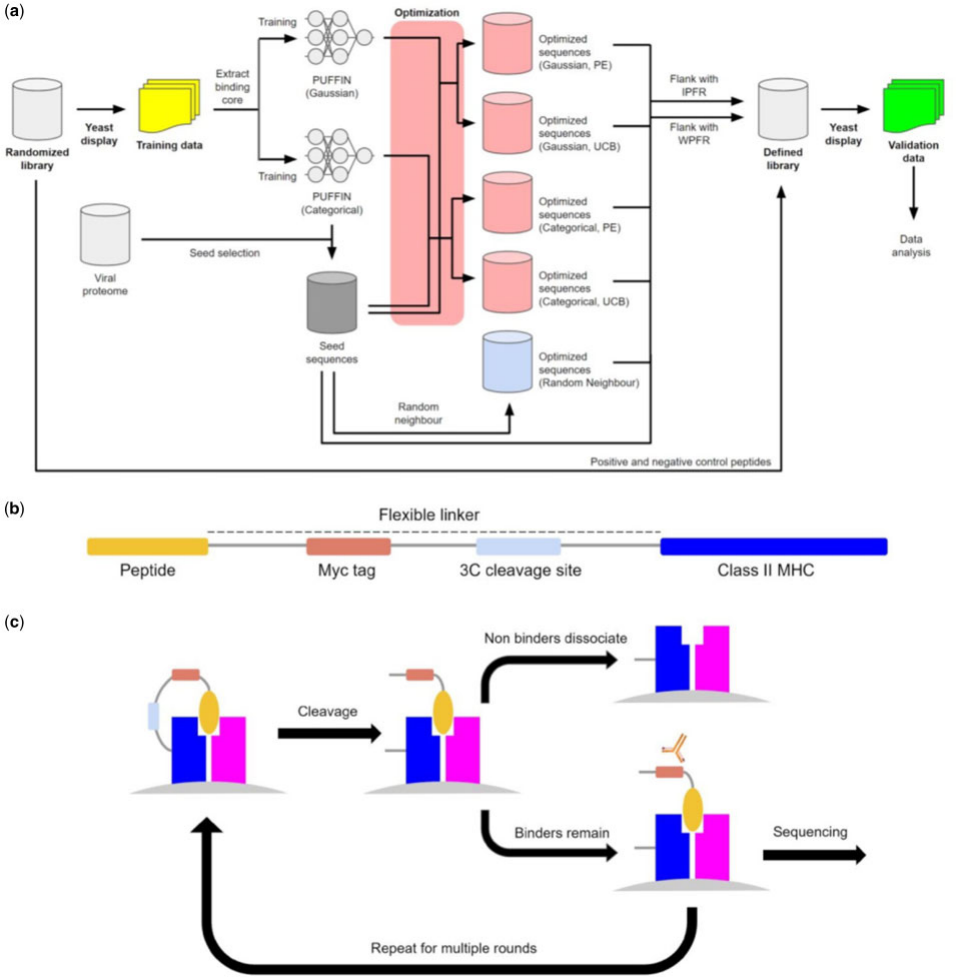
Characterization of peptide-MHCII binding by yeast display. (**a**) Details the generation of the training and validation data. The initial randomized library is used to generate the training data. The training data are used to train two variants of PUFFIN. One of these was used to select seeds from viral proteomes. These seeds were then optimized and combined with some peptides from the original randomized library to produce the defined library. Yeast display was done on the defined library to produce the validation data, on which most of our analysis is done. (**b**) Is a schematic of the construct used in the yeast display assay. (**c**) The overall process for yeast display. First, the peptide-MHC is expressed on the surface of yeast, and then the linker between peptide and the MHC molecule is cleaved. Peptide exchange is catalyzed, and yeast are selected which retain the Myc epitope tag. The resulting population is then sequenced and carried on to the next round

The overall study is illustrated in Figure 1a. We utilize peptide-MHC binding data from a yeast display platform for training PUFFIN (Rappazzo *et al.*, 2020) and adapt the platform for testing our optimized sequences (Fig. 1b and c). In this platform, MHCIIs are covalently linked to a query peptide with a flexible linker which contains a 3C protease cleavage site. When the linker is cleaved, unbound peptides can be displaced from the MHC in the presence of a high-affinity competitor peptide. The linker also contains a peptide-proximal epitope tag, which we use to enrich yeast that maintain peptide-MHC binding. Figure 1b illustrates the yeast display construct. Data are collected over multiple iterative rounds of selection. After each round of selection, deep sequencing is carried out on the enriched yeast and we extract the peptide identity from the sequencing data (Chao *et al.*, 2006; Magoč and Salzberg, 2011; Masella *et al.*, 2012). We filter the deep sequencing results for reads that match the invariant portions of the construct, from which we extract the peptide sequence. The resulting dataset assigns to every observed peptide its read count for each round of the yeast display assay.

We utilized data from two MHCII alleles: HLA-DR401 (HLA-DRA1*01:01, HLA-DRB1*04:01) and HLA-DR402 (HLA-DRA1*01:01, HLA-DRB1*04:02). This ensures that our results are not an allele-specific artifact and allows us to study optimization for multiple alleles.

For training the ML model, we utilized enrichment data from a library consisting of 108 random 9-mer peptides flanked by invariant peptide flanking residues (IPFR) which encourages binding in a single register and simplifies identification of anchor residues (Rappazzo *et al.*, 2020). This dataset contains orders of magnitude more peptide binders than comparable approaches and can be used for improved peptide affinity prediction (Rappazzo *et al.*, 2020), which will be beneficial when scoring and selecting optimized sequences. We used these data to train two predictors for each allele. Adam was used for optimization (Kingma and Ba, 2015) and dropout was used for regularization (Srivastava *et al.*, 2014). The first predictor models the enrichment as a continuous value and outputs a Gaussian distribution, while the second predictor models the enrichment as categoricals and outputs a probability distribution over the categories. In both cases, the enrichment value of a given 9-mer is based on the last round of its appearance in the yeast display experiment.

For testing our optimizations, we adapted the yeast display library for user-defined peptide libraries. This second yeast display library was cloned using a defined oligonucleotide library, and selections were repeated to enrich for peptide binders.

Both the yeast display assay and the predictor training process are described in greater detail in Supplementary Material.

### 2.3 We optimized the anchor residues of sequences drawn from viral proteomes

We proposed anchor optimizations to 9-mers drawn from the proteomes of the Zika, HIV and Dengue viral proteomes, which we refer to as seed sequences. We selected three sets of sequences on which to evaluate three different optimization tasks. These sets of sequences are:

1. 82 seed sequences that have some affinity for HLA-DR401, which we optimize for affinity to HLA-DR401.
2. 87 seed sequences that have some affinity for HLA-DR402, which we optimize for affinity to HLA-DR402.
3. 44 seed sequences that have high affinity for HLA-DR402 and some affinity for HLA-DR401, which we optimize for affinity to both MHC alleles.

We use predictions from the categorical ML predictor as a surrogate for affinity in this context. Specific details underlying seed selection is given in Supplementary Material.

PUFFIN was designed to characterize the uncertainty of its predictions by outputting a variance. This allows us to use various Bayesian acquisition functions as our objectives. For this study, we chose to study point estimate (PE) which is just the enrichment, and upper confidence bound (UCB) which adds the enrichment and the standard deviation of the prediction. For our third task of optimizing for both alleles, the objectives were computed for each allele individually and then added to produce the combined objective.

Optimizations using PE and UCB were performed with both the Gaussian and categorical models. Optimization with a given objective was done by enumerating and evaluating all possible anchor substitutions with that objective and selecting the top 10 scoring substitutions, giving a total of 40 optimized sequences for each seed. For each seed, 10 random anchor substitutions were also generated as a random control. We add these sequences to a new yeast display library for testing our designs.

Instead of adding the 9-mers directly, we first flanked them with IPFR so the sequences would resemble those from the original randomized library. Therefore, the sequences will take the form ‘AAXXXXXXXXXWEEG’, where ‘X’ denotes any residue (Rappazzo *et al.*, 2020). As a further control, we also flanked the 9-mers with their wild-type peptide flanking residues (WPFR), which were defined as the three residues that flanked the seed 9-mer in the source proteome. Finally, we sampled some sequences that performed well and some sequences that performed poorly in the training data and added them as positive and negative controls, respectively. We constructed a new yeast display library composed of these optimized peptides and controls, and we conducted another series of selections to enrich for binders. Figure 1a depicts this process.

To compare affinities between given peptides, for each peptide we estimated the proportion of that peptide which survives between rounds. This value is determined by fitting a geometric progression to the concentration of each peptide. We assume that read counts are drawn from a Poisson distribution that is parametrized by the concentration of the peptide multiplied by a constant that is dependent on the overall population being sequenced, and we fit the maximum likelihood estimate.

By fitting a geometric progression, we can extract the proportion between successive values in the progression, which we can interpret to be the proportion of peptides that survive a single round of the experiment. The constant of the sequenced population is undetermined, so the proportion we extract is a scalar multiple of the true proportion. Therefore, this proportion is unnormalized, so we refer to it as a round survival rate (RSR). While RSR is not unique, we find that it is able to provide a highly consistent ranking to the peptides (Supplementary Fig. S7).

This process is described in greater detail in Supplementary Material. We use RSR as a surrogate for affinity for the remainder of this text.

## 3 Results

### 3.1 Optimization improves the RSR of peptides for all optimization objectives

**Fig. 2.**
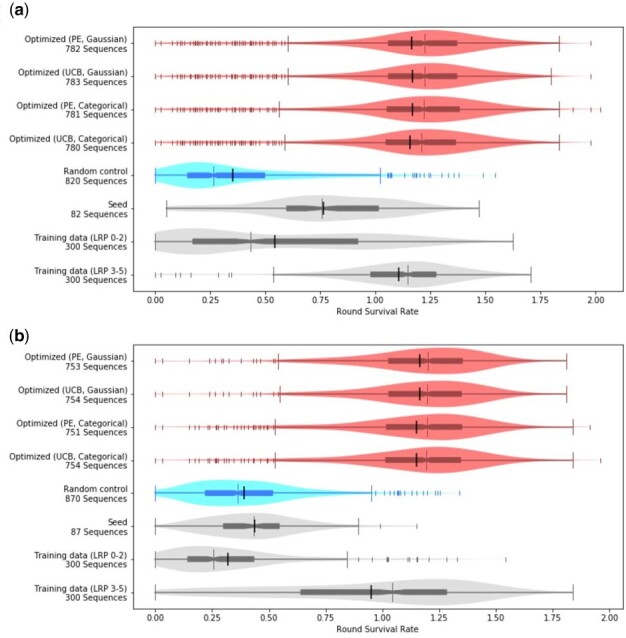
Anchor optimization improves RSR. The distributions of RSR for (**a**) HLA-DR401 and (**b**) HLA-DR402 are plotted for the optimized and control groups. The sequences from the training data are split into two groups: ‘initial experiment (LRP 0-2)’ is composed of sequences which did not appear after round 2 in the initial display assay and is shown as a negative control, while ‘initial experiment (LRP 3-5)’ is composed of sequences that did appear after round 2 and is shown as a positive control. The differences between the optimized groups and the negative controls [random control, seed and initial experiment (LRP 0-2)] are significant for both alleles, with  P≤1.58e-23 under the two-sided Mann–Whitney *U* test. Each plot is a combination of a box plot and a violin plot, where the distribution is shown by the violin plot in a lighter color, and the box plot shows the middle quartiles in a darker color along with the median. The mean is indicated by a black vertical line. Flier points are marked with the ‘|’ symbol

We first examine the overall RSR distribution of the following groups of sequences for each allele: sequences optimized for that allele with PE under the Gaussian model, UCB under the Gaussian model, PE under the categorical model, UCB under the categorical model, sequences with random anchor mutations (negative control), seed sequences, sequences from the training data which were not present after round 2 (negative control), and sequences from the training data which were present after round 2 (positive control). We find that the groups of optimized sequences exhibit higher RSRs for the alleles they were optimized for than either of the negative controls (Fig. 2). The improvements are statistically significant, with  P≤1.58e-23 between any optimized set and negative control for either allele by the two-sided Mann–Whitney *U* test.

We find that no optimization method significantly outperforms the others. Between any two groups of optimized sequences, *P*≥0.4 under the two-sided Mann–Whitney *U* test for both alleles. This can be explained by the overlaps observed in the optimizations proposed by different methods. When optimizing for HLA-DR401 affinity, if we compare the proposals generated by two optimization methods there are at most 2 seed sequences out of 82 whose proposed optimizations did not include any common sequences. Likewise, for HLA-DR402 for any two optimization methods there are at most 3 seed sequences out of 87 that had no overlap. This suggests that the specific objective does not significantly affect the quality of proposals. We compared the consistency of point estimate optimization proposals where uncertainty estimates are not required between NetMHCIIPan 4.0 (Reynisson *et al.*, 2020) and PUFFIN. We found that they proposed overlapping optimized sequences (Supplementary Fig. S10).

### 3.2 Sequences can be optimized for single or multiple alleles simultaneously

Since our method of generating proposals performs comparably under the different objectives we tested, we will focus the rest of our analysis on point estimate optimization under the Gaussian model for simplicity. We include an analogous analysis of the other optimization methods, which are similar, in Supplementary Material (Supplementary Figs S1–S4).

**Fig. 3.**
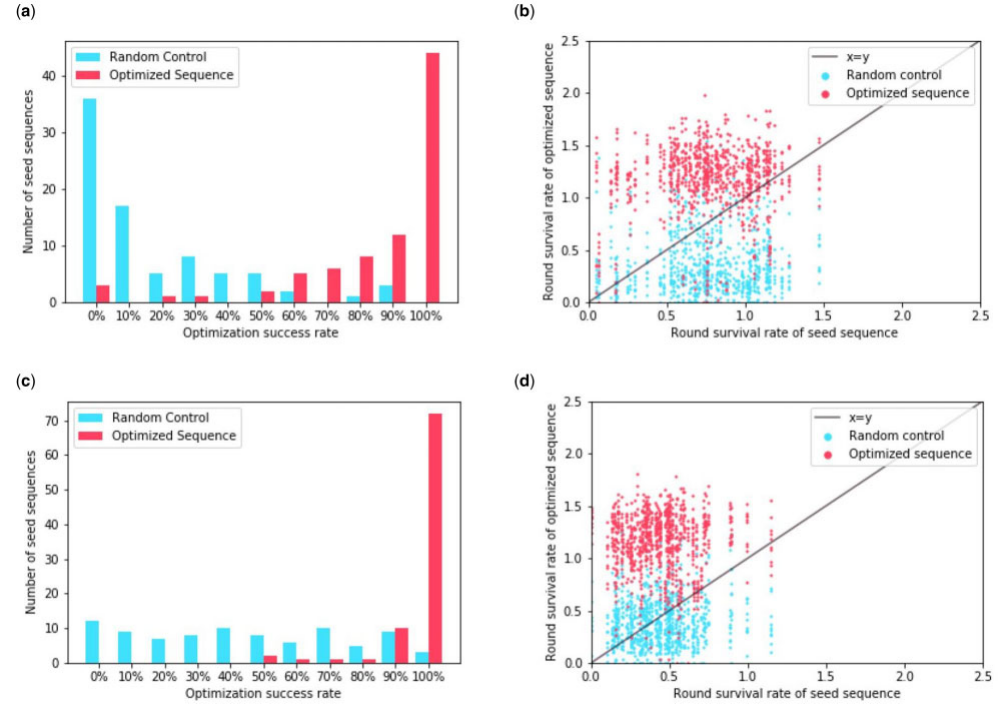
Number of sequences that exhibit improvement after optimizing with the point estimate objective under the Gaussian model. (**a**) For each seed sequence, we calculate the number of proposed optimizations that achieve an RSR for HLA-DR401 that is higher than that of the seed. We then take that as a percentage of the number of proposals to obtain the optimization success rate. We plot the distribution of these rates for both sequences optimized for HLA-DR401 affinity and the randomly perturbed sequences. (**b**) For each sequence optimized for HLA-DR401 affinity and randomly perturbed sequence, we plot their RSR for HLA-DR401 against the RSR of the seed sequence they derive from. (**c**) We calculate the distribution of optimization success rates for sequences optimized for HLA-DR402 using RSR for HLA-DR402. (**d**) We plot the RSR for HLA-DR402 of sequences optimized for HLA-DR402 against the RSR of their seed sequence

We find that most optimized sequences outperform their unaltered seed sequences (Fig. 3a and c). For HLA-DR401, for 44 out of the 82 seed sequences, all of the proposed optimizations performed better, while for HLA-DR402 this was the case for 72 out of the 87 seed sequences. In sequences where optimization was less effective, we find that generally the seed sequence already performs well via RSR (Fig. 3b and d).

**Fig. 4.**
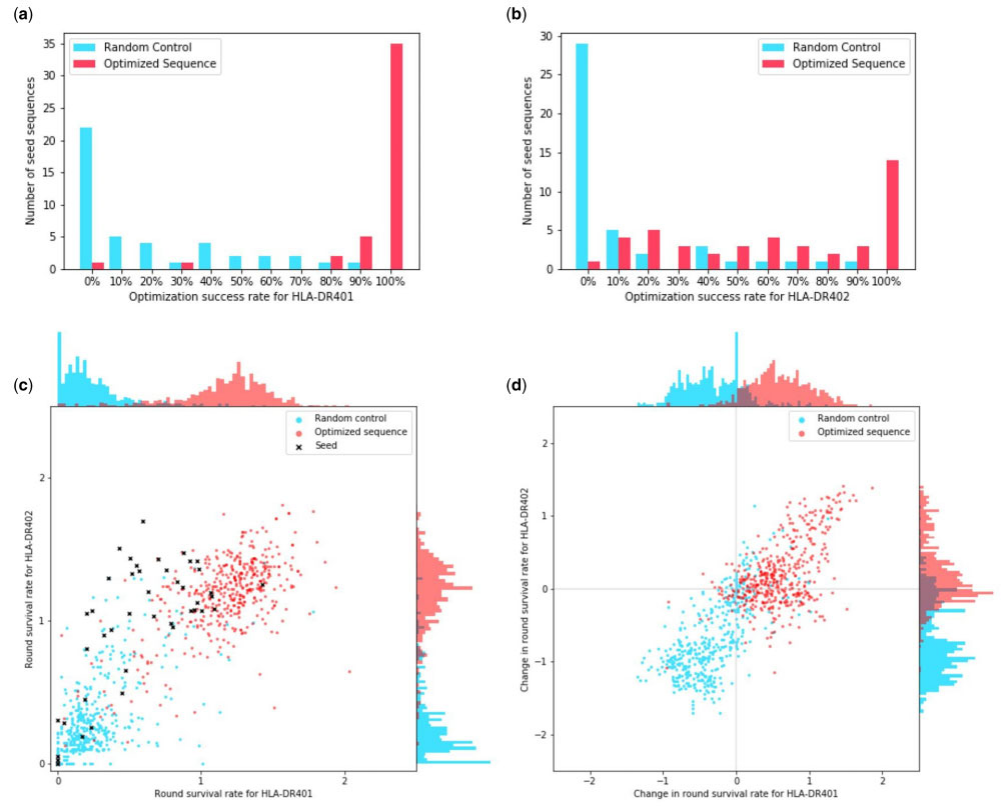
Number of sequences that exhibit improvement for multiple alleles after optimizing with the point estimate objective under the Gaussian model. (**a**) For each seed sequence, we calculate the number of proposed optimizations that achieve an RSR for HLA-DR401 that is higher than that of the seed. We then take that as a percentage of the number of proposals to obtain the optimization success rate. We plot the distribution of these rates for both sequences optimized for HLA-DR401 and HLA-DR402 affinity and the randomly perturbed sequences. (**b**) We produce the same distribution but with optimization success rates based on HLA-DR402 affinity. The seed sequences were selected to have high HLA-DR402 affinity. (**c**) For each optimized, random control and seed sequence, we plot their RSR for both alleles. (**d**) For each optimized and random control sequence, we take their RSR and subtract the RSR of the seed sequence they derive from to obtain the changes in their RSR

We find that sequences that were optimized for both alleles were generally able to improve their RSR for HLA-DR401 while maintaining their RSR for HLA-DR402 (Fig. 4). Out of a total of 44 seed sequences, there were 35 in which all proposed optimizations had a higher RSR for HLA-DR401. For 23 seed sequences, all proposed sequence optimizations outperformed the seeds on HLA-DR401 and achieved greater than 80% of the seed sequence RSR for HLA-DR402. For 13 seed sequences, all proposed optimizations outperformed the seeds on both HLA-DR401 and HLA-DR402.

If we instead consider seeds where the optimization criterion was reached for at least 8 out of the 10 proposed sequences, these values rise to 42, 35 and 18, respectively. For the random controls, they are 2, 1 and 1 (Fig. 4).

### 3.3 Our optimizations capture complex interactions between residue positions

**Fig. 5.**
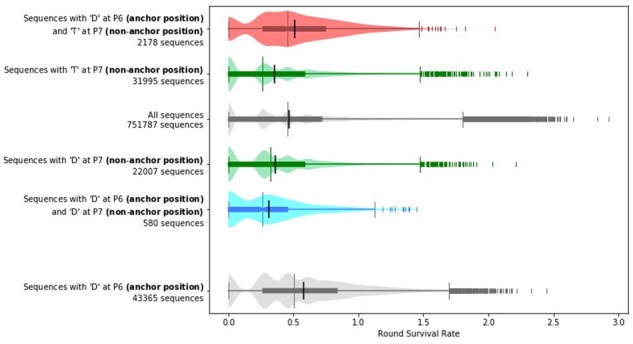
Whether a given anchor residue improves affinity can depend on non-anchor residues. Peptides with aspartic acid at P7 tend to have lower RSRs when compared to all peptides, and peptides that additionally have another aspartic acid at anchor position P6 tend to have even lower RSRs than the peptides that just have an aspartic acid at P7. In contrast, although peptides with threonine at non-anchor position P7 also tend to have lower RSRs when compared to all peptides, peptides that additionally have an aspartic acid at anchor position P6 tend to have higher RSRs instead even when compared to all peptides. The differences found in these comparisons are significant, with  P≤4.968e-5 between any two groups mentioned above under the Mann–Whitney two-sided *U* test. The sequences plotted and used for computing significance are from the training data. Each plot is a combination of a box plot and a violin plot, where the distribution is shown by the violin plot in a lighter color, and the box plot shows the middle quartiles in a darker color along with the median. The mean is indicated by a black vertical line. Flier points are marked with the ‘|’ symbol

By analyzing our training data, we find that the identity of residues outside of the primary anchor residues can have a significant impact on which anchor residues will improve affinity. As an example (Fig. 5), for HLA-DR401 if a sequence contains a threonine (T) at the non-anchor position P7, then having an aspartic acid (D) at anchor position P6 tends to increase the RSR. However, if the sequence contains a D at the non-anchor position P7, then having a D at P6 tends to decrease the RSR instead. Higher order effects can be seen between other anchor and non-anchor positions as well, so these relationships are not limited to adjacent positions nor to residues at P7, which can be considered an auxiliary anchor because of its contacts with the MHC groove (Jones *et al.*, 2006).

The dependency between anchor positions and non-anchor positions can be observed in the proposals generated by our method. Out of the 820 sequences proposed using PE under the Gaussian model for HLA-DR401, 20 (2%) have a D at anchor position P6. Six of our seed sequences have a T at P7; of our proposed optimizations for these seeds, 17/60 (28%) have a D at P6. Conversely, nine of our seed sequences have a D at P7, and none of their 90 proposed optimizations have a D at P6. This demonstrates the advantages of enumerating the full anchor residue landscape as it allows the capture of these higher order effects.

Given the presence of the higher order effects between peptide positions, including non-anchor positions, it seems unlikely that a more naive approach to anchor optimization could be as successful. In particular, it is unlikely that there exists a set of anchor residues that would optimize affinity in all non-anchor contexts. As further support for this, we find that there are no sets of anchor residues that were proposed for all seed sequences for any optimization task, even when combining the proposed optimizations across all four of our optimization methods. For HLA-DR401 optimization, the most frequently proposed set is Y, D, T, A at anchor positions P1, P4, P6, P9 (respectively), which was proposed for 54 out of the 82 seeds. For HLA-DR402, the most frequently proposed set is L, W, T, A at P1, P4, P6, P9 (respectively), which was proposed for 44 out of the 87 seeds. For optimization for both alleles, the most frequently proposed set is F, M, N, A at P1, P4, P6, P9 (respectively), which was proposed 34 out of 44 times.

### 3.4 Optimized sequences outperform seed sequences in the absence of the invariant flanking residues, but is less effective

All the optimized peptides we have presented so far have been flanked with IPFR, which were used to train the model. If we replace the IPFR with WPFR, we observe that the optimized sequences still outperform the seed and random controls (Supplementary Fig. S6). The improvement is still significant, with  P≤5.51e-5 when comparing the optimized sequences to the random control or seed sequences for either allele under the two-sided Mann–Whitney *U* test. However, the optimized sequences with WPFR significantly underperform their IPFR counterparts (*P*≤1.36e-22 for either allele under the two-sided *U* test).

The reduction in improvement is only observed in the optimized sequences for WPFR. In the case of the seed and random control groups, the WPFR sequences either do not display any significant difference or mildly outperform the IPFR counterparts (0.0018≤P≤0.92 under the two-sided Mann–Whitney *U* test).

### 3.5 Optimized sequence motifs are consistent with MHC binding preferences

**Fig. 6.**
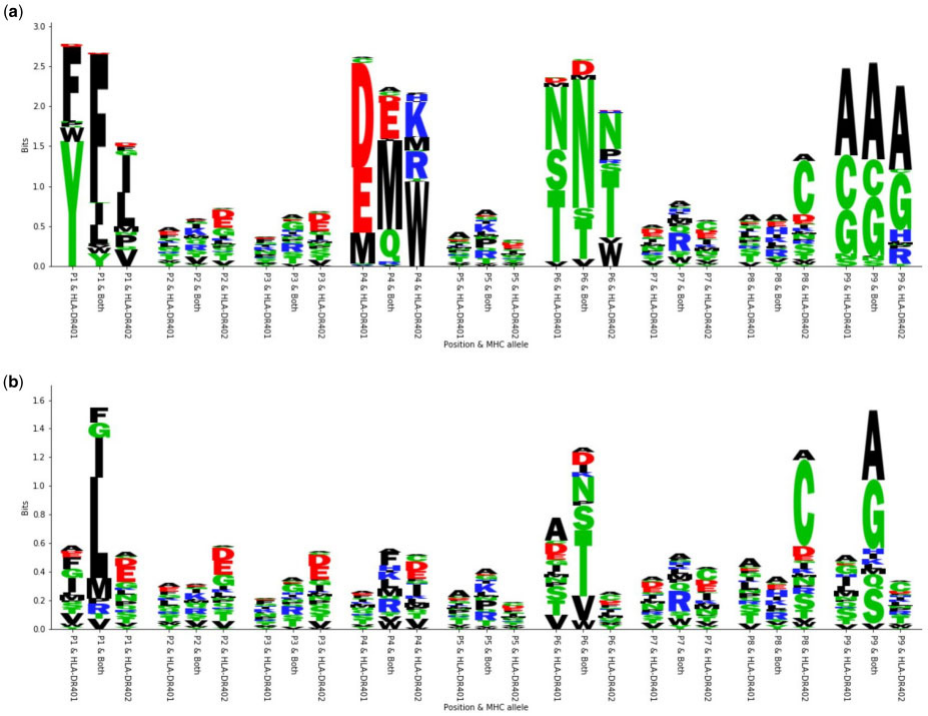
Motifs arising from optimization with the PE objective under the Gaussian model. (**a**) Sequence logos depicting the motifs present in the optimized sequences. For each position, three different residue distributions are shown. The first one shows the distribution for the sequences optimized for HLA-DR401, the last one shows the distribution for the sequences optimized for HLA-DR402, and the one in the middle shows the distribution for the sequences optimized for both alleles. Sequence logos were generated with a custom script. (**b**) Sequence logos depicting the motifs present in the original seed sequences for comparison with the same setup

The peptide optimizations made by our machine learning models are consistent with the structures and peptide-binding motifs of HLA-DR401 and HLA-DR402 in our training data. The polymorphisms between HLA-DR401 and HLA-DR402 affect the P1 and P4 binding pockets. Both alleles prefer hydrophobic amino acids in the P1 pocket, although HLA-DR401 prefers larger amino acids, while the truncated HLA-DR402 pocket prefers smaller amino acids. In the P4 pocket, HLA-DR401 prefers acidic residues, and HLA-DR402 prefers basic residues and large hydrophobic residues. The conserved P6 and P9 binding pockets prefer polar and small amino acids, respectively. The preference for each allele is reflected in MHC allele-specific peptide optimization, shown for the optimization with PE objective under the Gaussian model as an example (Fig. 6a). Joint MHC optimization is also consistent with these preferences: P1 and P4 amino acids are mutually preferred between both alleles, such as F/I/L at P1 and increased usage of M at P4. P6 and P9 amino acids are consistent with usage in individual allele-optimized peptides. Amino acid frequency in the seed sequences is also shown for reference (Fig. 6b).

## 4 Discussion

In this work, we introduced our method for optimizing the affinity of peptide sequences for MHCIIs by replacing their anchor residues with more optimal residues generated with the help of a machine learning model. We validated this technique on two different MHCII alleles, and showed that it is possible to optimize a single sequence for multiple alleles simultaneously. We have developed a high throughput yeast display-based pipeline to test our optimized sequences, and we introduced the notion of an RSR which allows us to compare the results of the assay.

We demonstrated that our method leverages deep learning models in a way that allows the proposed optimizations to capture complex interactions between residues. Our ability to optimize sequences for two alleles simultaneously suggests that the method can generalize to even more complicated objectives. These contributions improve our ability to engineer peptides for therapeutic purposes, and allows us to develop more robust cocktails by allowing their constituent peptides to fulfill multiple objectives.

We note that the method for generating proposals is independent of the specific predictor used. Our results indicate that taking uncertainty into account does not significantly improve the quality of the proposals over using a simple point estimate, so a model that quantifies its uncertainty is not strictly necessary. Therefore, substituting PUFFIN for another algorithm that performs comparably to PUFFIN should yield similar results.

As a caveat, we note that our optimization is less effective if we allow arbitrary flanking residues. The reduction in improvement when we change from IPFR to WPFR is only observed in the optimized sequences and is not observed in seed or sequences with random anchor residues. Therefore, it is likely that the drop in performance is due to the predictor being trained on IPFR data, so the predictor is unable to take the effects of flanking residues or register shifts into account. The IPFRs also contain preferred amino acids in the flanking sequences, such as the tryptophan at position P10. Aromatic residues at P10 have been shown to bolster binding and may impact the superior performance of IPFR peptides compared to WPRF peptides (Rappazzo *et al.*, 2020; Zavala-Ruiz et al., 2004). As noted above, since our method is independent of the specific underlying predictor, we should be able to address this issue by replacing our current predictor with one that takes the flanking residues into account. More generally, the quality of our optimization should improve as the quality of predictors available continues to improve.

Our future work will extend our method to incorporate wild-type flanking residue information in our optimization, and will seek to characterize the effect of anchor optimization on peptide immunogenicity.

## Funding

This work was supported in part by a Schmidt Futures grant to D.K.G. and M.E.B., a Melanoma Research Alliance Grant to M.E.B. and the National Institutes of Health (R01CA218094 to D.K.G. and P30CA14051 to M.E.B.). Siddhartha Jain’s contribution was made prior to him joining Amazon.

*Conflict of Interest*: Michael E. Birnbaum is an advisor to Repertoire Immune Medicine. David K. Gifford is a founder of ThinkTx.

## Data availability

The data underlying this article are available in Github, at https://github.com/gifford-lab/MHC2-optimization.

## Supplementary Material

btab131_Supplementary_Data
