# Supplementary material for: Machine learning optimization of peptides for presentation by class II MHCs
Source: Bioinformatics. 2021 Mar 11;37(19):3160–7. doi: 10.1093/bioinformatics/btab131 (PMC8504626; doi:10.1093/bioinformatics/btab131)
Supplement: btab131_Supplementary_Data [file btab131_supplementary_data.pdf]

Supplementary material

Collecting enrichment data using a high throughput peptide display assay

We utilize peptide-MHC binding data from a yeast display library of ∼ 10^8 random 9-mer peptides (Fig. 1)(Rappazzo et al., 2020). The peptides are flanked by invariant peptide flanking residues (IPFR), which encourages binding in a single register and simplifies identification of anchor residues. The IPFR consists of "AA" on the N-terminus and "WEEG" on the C-terminus. Paired-end sequencing reads (Rappazzo et al., 2020) were assembled via FLASH (Magoč and Salzberg, 2011) and filtered for correct length and 3C cut site sequence.

In order to test our optimized sequences, we adapted the yeast display platform and workflow from randomized peptide libraries to presentation of user-defined peptides. We designed a 36,000-member defined library containing our optimized sequences, which was synthesized by Twist Bioscience as a single-stranded oligo pool with a maximum length of 120 nucleotides. The oligo pool was amplified with low cycle number PCR then amplified with construct DNA using overlap extension PCR. This longer DNA product was assembled with the linearized pYal vector in yeast at a 5:1 mass ratio of insert:vector and electroporated into electrocompetent RJY100 yeast. To better assess enrichment, the HLA-DR401 and HLA-DR402 defined peptide libraries were doped into a ~20-million-member randomized peptide library containing stop codons at a ratio of approximately 1:500 so that each unique peptide was represented at similar starting frequency. The diverse null library had the peptide encoded as

"NNNTAANNNNNNNNNNTAGNNNNNNNNNNNNNTGANNNNNN", where N indicates any nucleotide. Doping into this library provides a null set of peptides over which real binders must enrich.

For each round of selection, yeast were washed into PBS, with competitor peptide (HLA-DR401: $HA_{306\text{-}318}$, 1uM; HLA-DR402: $CD48_{36\text{-}53}$, 5uM) and 1uM 3C protease, then incubated for 45min room temperature. After incubation, yeast were washed into cold acid saline (20mM pH5 citric acid, 150mM NaCl) with competitor peptide (same concentration as first incubation) and 1uM HLA-DM, then incubated overnight at 4°C. Negative selections for non-specific binders was performed with anti-AlexaFluor647 magnetic beads (Milltenyi Biotech; Bergish Gladbach, Germany), followed by a positive selection consisting of incubation with anti-Myc-AlexaFluor647 antibody (1:100 volume:volume) and positive selection with anti-AlexaFluor647 magnetic beads. The first round was conducted on 400 million yeast for 20x coverage of peptides and incubations were conducted in 2mL PBS and 4mL acid saline. For subsequent rounds, 25 million yeast were selected; incubations were conducted in 250uL PBS, 500uL acid saline. Four iterative rounds of selection were performed and repeated in duplicate. Between rounds, yeast were grown to confluence at 30°C in SDCAA (pH=5) yeast media and subcultured into SGCAA (pH=5) media at OD_{600}=1 for two days at 20°C (Chao et al., 2006).

Following selections, plasmid DNA was isolated from 10 million yeast from each round using a Zymoprep Yeast Miniprep Kit (Zymo Research; Irvine, CA). Amplicons were generated to capture the peptide through the 3C protease site. Unique barcodes were added for each library and round of selection and i5 and i7 anchors added through two

rounds of PCR. Amplicons were sequenced on an Illumina MiSeq (Illumina; San Diego, California) at the MIT BioMicroCenter, with a paired-end MiSeq v2 300nt kit.

Forward and reverse reads are assembled using PandaSeq (Masella et al., 2012). Data were processed using in-house scripts to extract peptide sequences with correctly encoded constant flanking regions. Peptides were filtered for exact matches to the defined sequences ordered from Twist and those matching the DNA encoding of the randomized null library.

HLA-DM was recombinantly expressed as previously described (Rappazzo et al., 2020). In brief, the ectodomains of the alpha and beta chains were followed by a poly-histidine purification site and encoded in pAcGP67a vectors. Plasmids for each chain were separately transfected into SF9 insect cells with BestBac 2.0 baculovirus DNA (Expression Systems; Davis, CA) and Cellfectin II reagent (Thermo Fisher; Waltham, MA). Cells were propagated to high virus titer, co-titrated to ensure an equal ratio of alpha and beta expression, and co-transduced into Hi5 cells. Following 48-72 hours of incubation, proteins were purified with Ni-NTA resin and purified with size exclusion chromatography on an AKTAPURE FPLC S200 increase column (GE Healthcare; Chicago, IL).

Training a neural network based ML model to predict the enrichment category of a peptide

We trained a neural network-based machine learning (ML) model (PUFFIN) (Zeng and Gifford, 2019) to predict the enrichment label of a new peptide. The predictor takes a 9 residue peptide sequence as input, and outputs an enrichment label. We use the final round

where a peptide is observed in the peptide display assay as our enrichment label for both training and prediction. For example, if a sequence appears in the sequencing reads for round 3 but fails to appear in round 4 or any other future rounds, it receives the label "3". To improve the granularity, round 5 presence was further split up into 3 categories, where "5" indicates round 5 presence with less than 10 read counts in round 5, "6" indicates round 5 presence with a read count between 10-99 inclusive, and "7" indicates round 5 presence with a read count of 100 or more. A label of 0 is given to sequences that only appear before any enrichment is performed. This gives a total of 8 enrichment categories.

PUFFIN is an ensemble of deep residual neural networks that is regularized by dropout and controlled for overfitting with validation data. Each component model consists of one convolutional layer, five residual blocks, and one output layer. Each residual fits the difference between the input and the output of a residual block with two convolutional layers. Each convolutional layer has 256 convolutional filters and is followed by a batch-norm layer. ReLU is used as non-linearity throughout the network.

For each allele, we trained two predictors to predict the enrichment labels. The first predictor assumes the enrichment labels 0-7 are realizations of a continuous random variable taken from a Gaussian distribution, and was trained to output a mean and variance. The second predictor models the labels as categoricals, and outputs a discrete probability distribution over the 8 labels 0-7. For regularization, dropout (Srivastava et al., 2014) is used in the output layer with a dropout probability of 0.2. We randomly hold out 10% of the data for validation, and the rest is used for training. We use Adam (Kingma and Ba, 2015) to minimize the negative log-likelihood of the observed enrichment under the

probability distribution parameterized by the output of the neural network. We train for 50 epochs and select the model from the epoch where validation loss is minimized.

While the outputs of each predictor naturally characterize aleatoric uncertainty, we also characterize the epistemic uncertainty through ensemble methods (Lakshminarayanan et al., 2017). Specifically, we generate 10 training and validation splits of our data and train 2 separate predictors for each split, giving us an ensemble of 20 predictors. When performing predictions, we run each predictor 50 times with dropout turned on (Gal and Ghahramani, 2016), resulting in a total of 1000 predictions for each input. The final output is then characterized by a mean and variance, where the mean is the average of the distribution means over all 1000 trials, and the final variance is the average of the distribution variances for each trial plus the variance of the distribution means.

Running these predictors on sequences from the defined library show that these variants of the PUFFIN model obtain state of the art predictions (Supplemental Figure 5). We also ran PUFFIN predictions on a published orthogonal 13-mer peptide yeast display test set (Rappazzo et al., 2020), to compare to existing models. We observe comparable performance between PUFFIN and current state of the art (area under the receiver operator characteristic curve of 0.91, 0.91, and 0.82, for Gaussian PUFFIN, Categorical PUFFIN, and NetMHCIIpan4.0 EL score, respectively, on a dataset with a 1:1 ratio of binders to non-binders; and positive predictive values, the fraction of binders observed in the predicted top 5% of predictions, of 0.57, 0.57, and 0.29, for Gaussian PUFFIN, Categorical PUFFIN, and NetMHCIIpan4.0 EL score, respectively, on dataset with 1:19 ratio of binders

to non-binders), where PUFFIN-identified cores are the maximum-scoring 9-mer within the 13-mer.

Using an ML model to compute an objective function for anchor optimization

For both the Gaussian and the categorical predictors, we considered two different objective functions for scoring 9-mers: point estimate (PE) and upper confidence bound (UCB). In both functions, we first run our predictor over the 9-mer to obtain a predicted mean and variance. Then to compute the PE objective, we simply return the mean. To compute UCB, we return the sum of the mean and the standard deviation, which we take to be the square root of the variance.

This gives us a total of 4 methods for scoring 9-mers. For each method, given an input 9-mer to optimize, we enumerate all possible residue substitutions at positions 1, 4, 6, and 9 (sequences are 1-indexed). For each substitution, we compute its score using our objective function, and in the end we output the 10 sequences that score the highest as proposed optimizations.

Designing a validation library to test the efficacy of anchor optimization

We tested the efficacy of anchor optimization on three tasks as outlined in the main body of the paper. Our evaluation was conducted using viral peptides selected from the Zika, HIV, and Dengue viral proteomes. The 9-mers in the candidate proteomes have no overlap with the peptides in our random peptide training library. We selected seeds for optimization from Zika, HIV, and Dengue based on the predictions of the categorical predictor. We first filter the sequences by removing all whose PUFFIN prediction has a predicted variance

higher than the median predicted variance. For the seeds for Task 1 and Task 2, we selected peptides with a predicted enrichment mean between 2 and 3, yielding 82 seeds for HLA-DR401 and 87 seeds for HLA-DR402. For the seeds for Task 3, we selected peptides with a predicted HLA-DR401 enrichment mean below 3 and a predicted HLA-DR402 enrichment mean above 5, resulting in 44 seeds.

For each seed sequence, we ran each of our 4 optimization methods over it for each allele, giving 10 optimized sequences for each method. As a control, we also proposed 10 random anchor residue mutations for each seed.

We then take the seed, optimized, and random sequences and flank them with IPFR. As a control, we also produce a second set of sequences from the same 9-mers but flanked with WPFR, defined to be the 3 residues that flank the original seed sequence in the original proteome. This forms the basis of the library.

As a further control, we added sequences from the original training data to the library. For each allele, we sampled 300 sequences that had no presence after the second round of selections, and 300 sequences that had presence after the second round of selections, giving us 1200 sequences overall.

Calculating round survival rate as a representation of enrichment

The enrichment information reported in the peptide display assay comes in the form of a vector of read counts indexed by round. In order to compare enrichment between different peptides, we assign to each peptide a value that can be interpreted as an unnormalized proportion of that peptide that survives between rounds of enrichment. We will refer to

this quantity as a round survival rate (RSR), where a higher RSR will be indicative of higher enrichment.

To calculate a peptide's RSR, we consider a simplified model where the peptide has a starting concentration drawn from a given prior, and the dominating event is peptide dissociation from the MHCII. Additionally, we assume that we can treat the entire experiment as though it was happening in one solution, and everything that occurs between rounds can be captured by a scaling factor. Finally, we suppose that read counts follow a Poisson distribution parameterized by the concentration multiplied by a scaling factor.

$$R_{S,i} \sim \text{Poisson}(a_i c_s p_S^{i})$$

$$\ln(c_s) \sim \text{Gaussian}(0,1)$$

For all peptides S and all rounds i, where $R_{S,i}$ is the read count of peptide S in round i, c_S is the starting concentration of the peptide S, p_S is an unnormalized proportion of peptide S that survive to the next round, and a_i is a round specific constant. The prior for constraining c_S is for regularization purposes, and a log normal distribution was selected for its interpretability as the result of geometric Brownian motion.

We then define the RSR for peptide S as the maximum a posteriori (MAP) estimate of p_S. This value is not unique, as an adequate scaling in the a_i values can give the same probabilities with different p_S values. However, such a transformation preserves the ratio between p_S, and in practice we find that the estimates converge reliably (Supplemental Figure 7). We estimate these values by iteratively optimizing each variable individually for

500 rounds. 23 rounds were carried out with random initializations, where p_S were drawn from Uniform(0.1,1) and $\ln(c_S)$ were drawn from Gaussian(0,1). We compute how well the model estimates the true read counts (Supplemental Figure 8).

For experiments conducted over the defined library, we use the null library to construct a baseline model where the read count in each round follows its own Poisson distribution. The lambda parameter for each distribution was estimated by average read counts (with added pseudocounts for peptides which don't show up in any round added to make the variance of the distribution in the zeroth round match the mean). When performing MAP estimation, an additional parameter is given to each peptide which indicates whether it comes from this baseline distribution or from the model described above to filter out noise.

RSR values of replicate selections of the defined library are concordant with the first replicate (Pearson and Spearman correlation coefficients 0.81-0.84; Supplemental Figure 9), suggesting selections and RSR determination is reproducible. A subset of sequences is absent from a single replicate due to stochastic dropout, which likely occurs in the initial rounds of selection when each member of the library is present at low frequency.

References

Chao, G., Lau, W. L., Hackel, B. J., Sazinsky, S. L., Lippow, S. M., and Wittrup, K. D. (2006). Isolating and engineering human antibodies using yeast surface display. Nature protocols, 1, 755–768.

Gal, Y. and Ghahramani, Z. (2016). Dropout as a bayesian approximation: Representing model uncertainty in deep learning. In International Conference on Machine Learning, pages 1050–1059.

Kingma, D. P. and Ba, J. (2015). Adam: A method for stochastic optimization. In International Conference on Learning Representations.

Lakshminarayanan, B., Pritzel, A., and Blundell, C. (2017). Simple and scalable predictive uncertainty estimation using deep ensembles. In Advances in Neural Information Processing Systems, 30, 6402–6413.

Magoč, T. and Salzberg, S. L. (2011). Flash: fast length adjustment of short reads to improve genome assemblies. Bioinformatics, 27, 2957–2963.

Masella, A. P., Bartram, A. K., Truszkowski, J. M., Brown, D. G., and Neufeld, J. D. (2012). PANDAseq: paired-end assembler for illumina sequences. BMC bioinformatics, 13, 31.

Rappazzo, C. G., Huisman, B. D., and Birnbaum, M. E. (2020). An unbiased determination of class ii mhc peptide repertoires via large yeast-displayed libraries. Nature Communications, 11, 4414.

Srivastava, N., Hinton, G., Krizhevsky, A., Sutskever, I., and Salakhutdinov, R. (2014). Dropout: a simple way to prevent neural networks from overfitting. The Journal of Machine Learning Research, 15, 1929–1958.

Zeng, H. and Gifford, D. K. (2019). Quantification of uncertainty in peptide-mhc binding prediction improves high-affinity peptide selection for therapeutic design. Cell Systems, 9, 159–166.

Supplemental Figure 1

a)

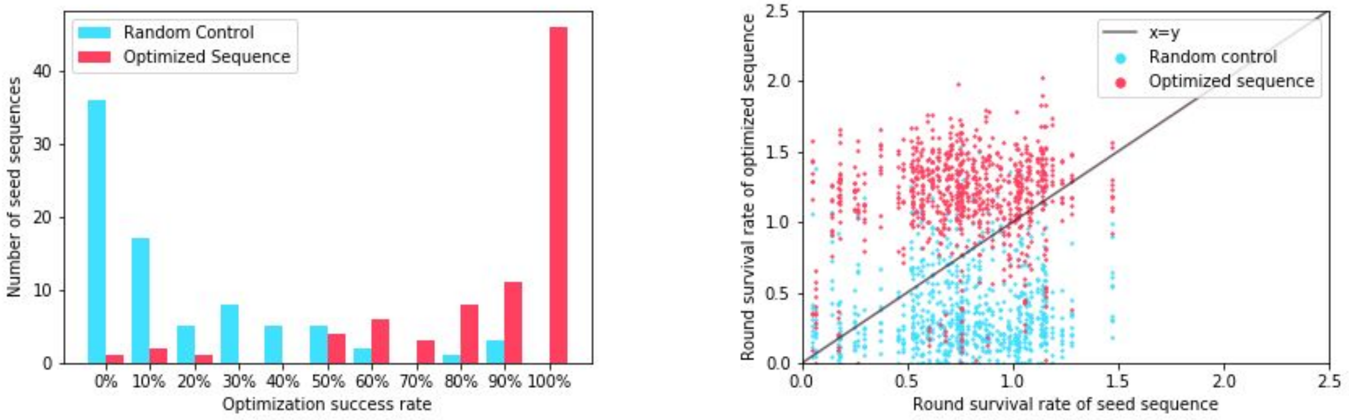

b)

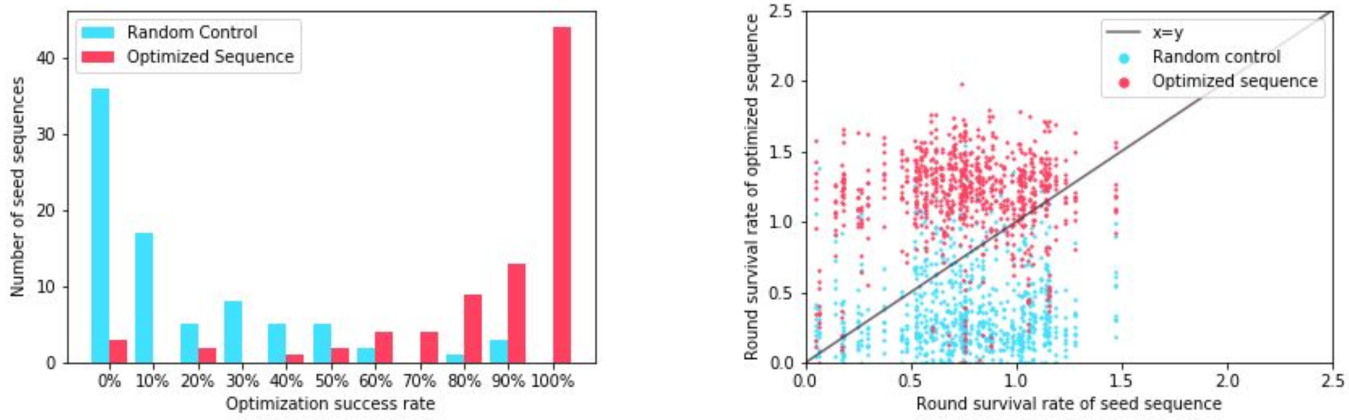

c)

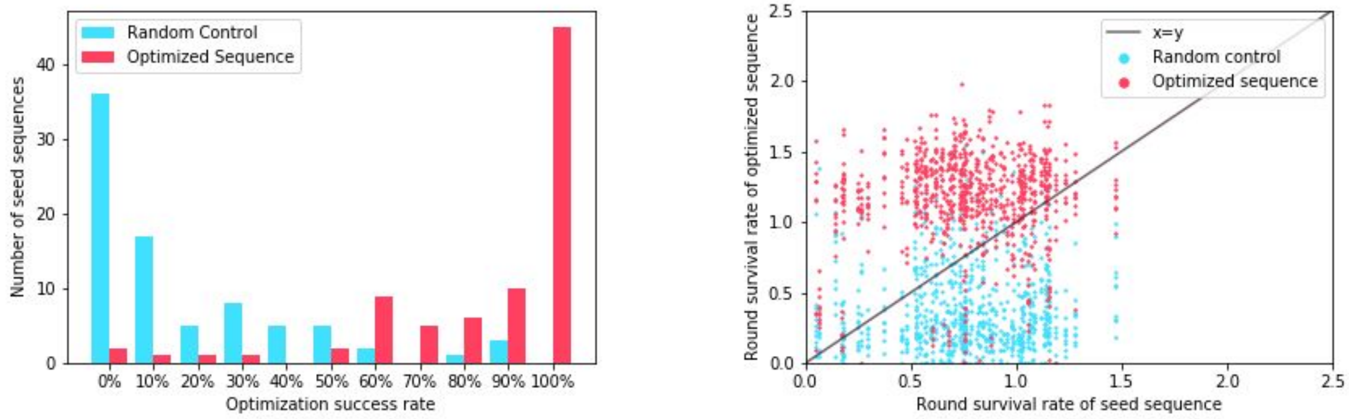

Number of sequences that exhibit improvement for other optimization methods in DR401. These depict the same plots as Figure 3, but for different optimization schemes for HLA-DR401. **a)** PE under the categorical model. **b)** UCB under the Gaussian model. **c)** UCB under the categorical model.

Supplemental Figure 2

a)

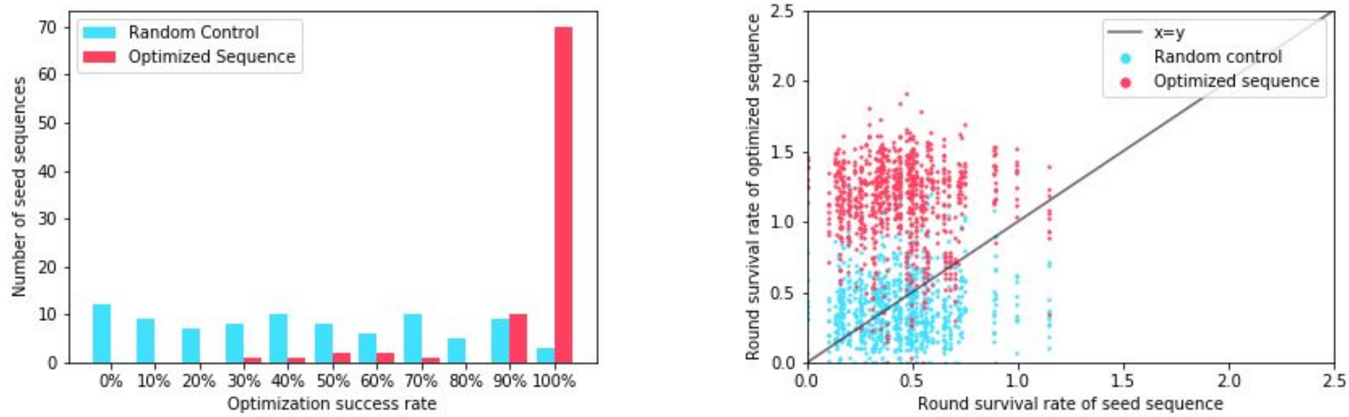

b)

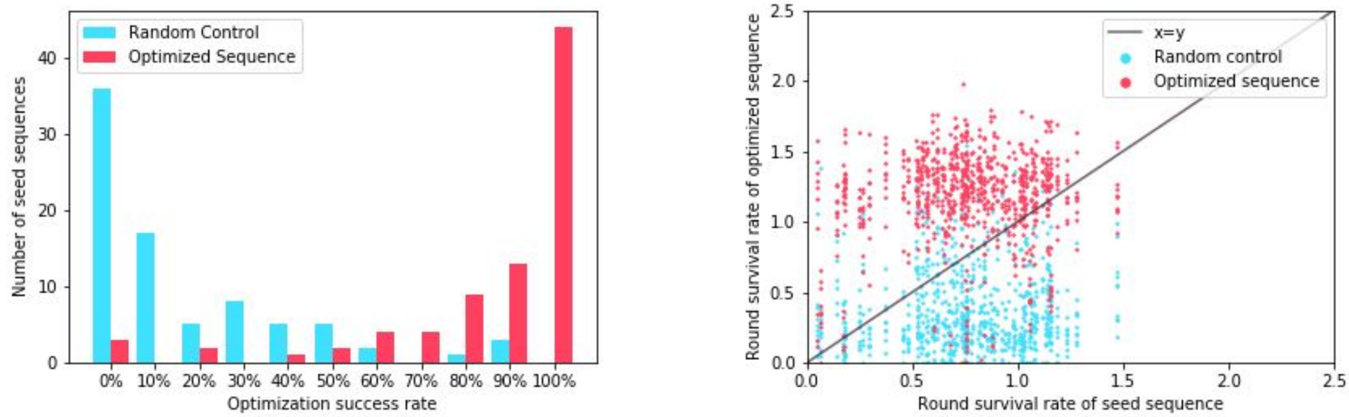

c)

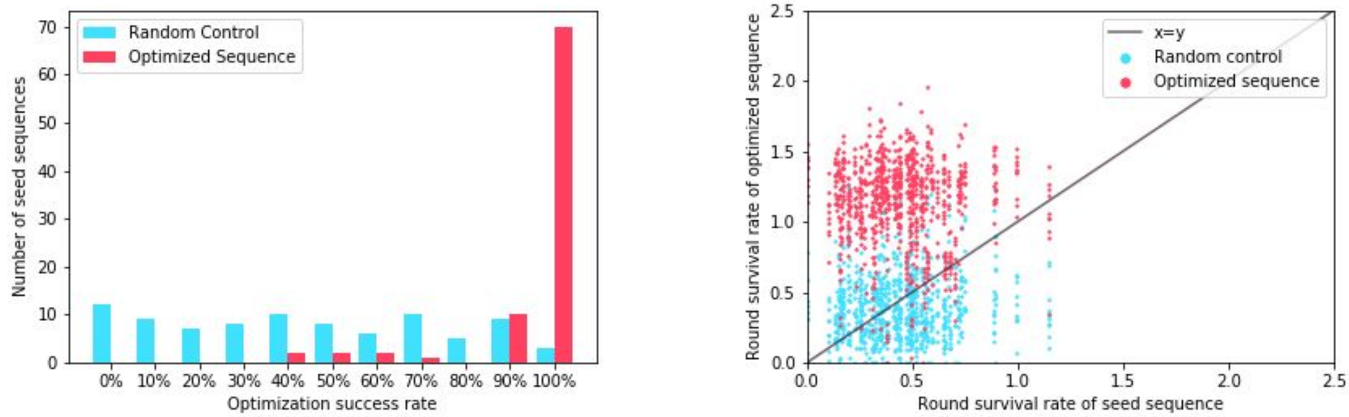

Number of sequences that exhibit improvement for other optimization methods in DR402. These depict the same plots as Figure 3, but for different optimization schemes for HLA-DR402. **a)** PE under the categorical model. **b)** UCB under the Gaussian model. **c)** UCB under the categorical model.

Supplemental Figure 3

a)

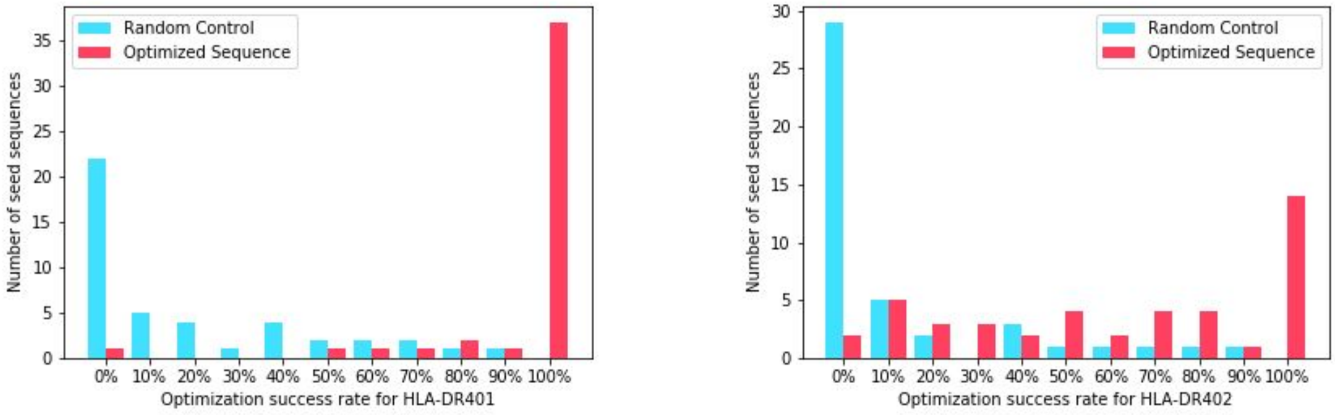

b)

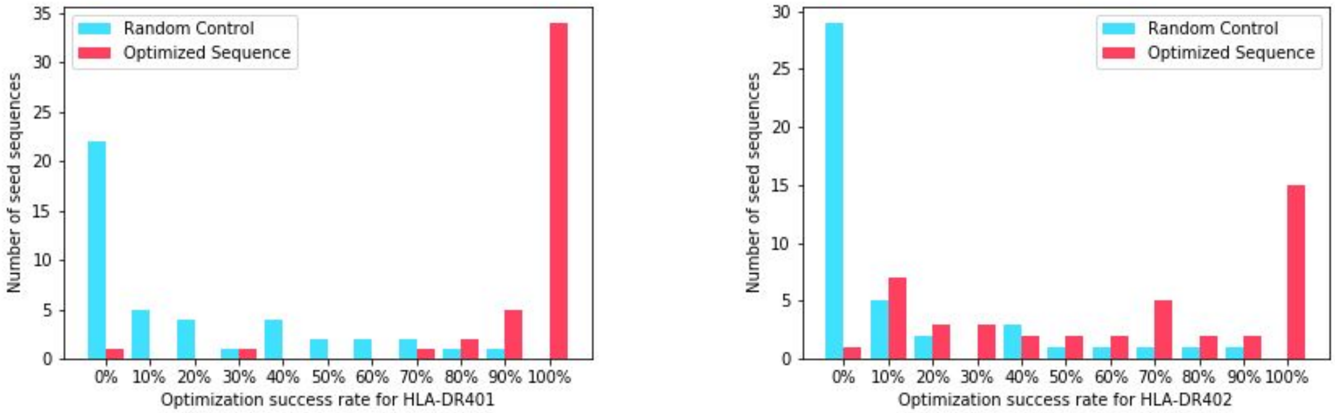

c)

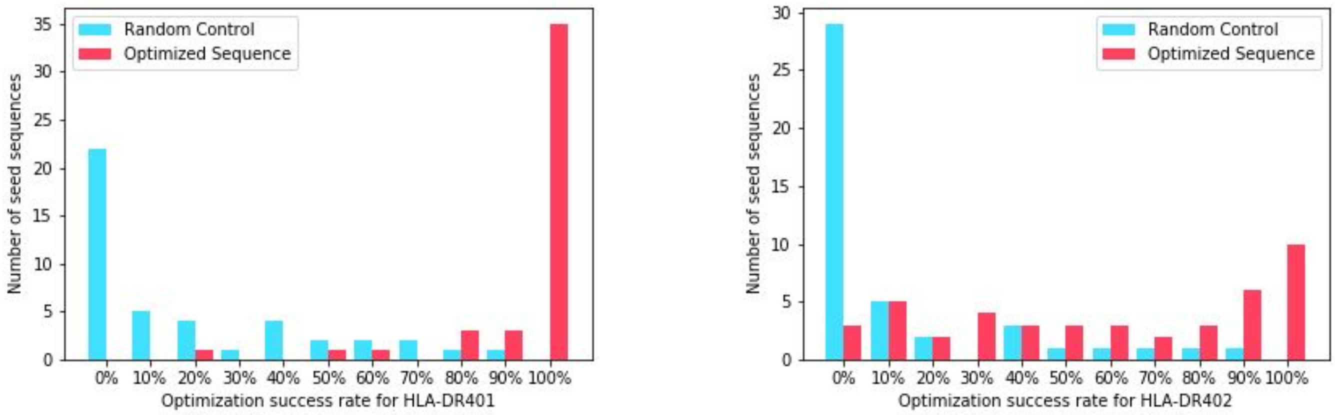

Multiple allele optimization improvements for other optimization methods. The same plots as Figure 4A/B, but for different optimization schemes. **a)** PE under the categorical model. **b)** UCB under the Gaussian model. **c)** UCB under the categorical model.

Supplemental Figure 4

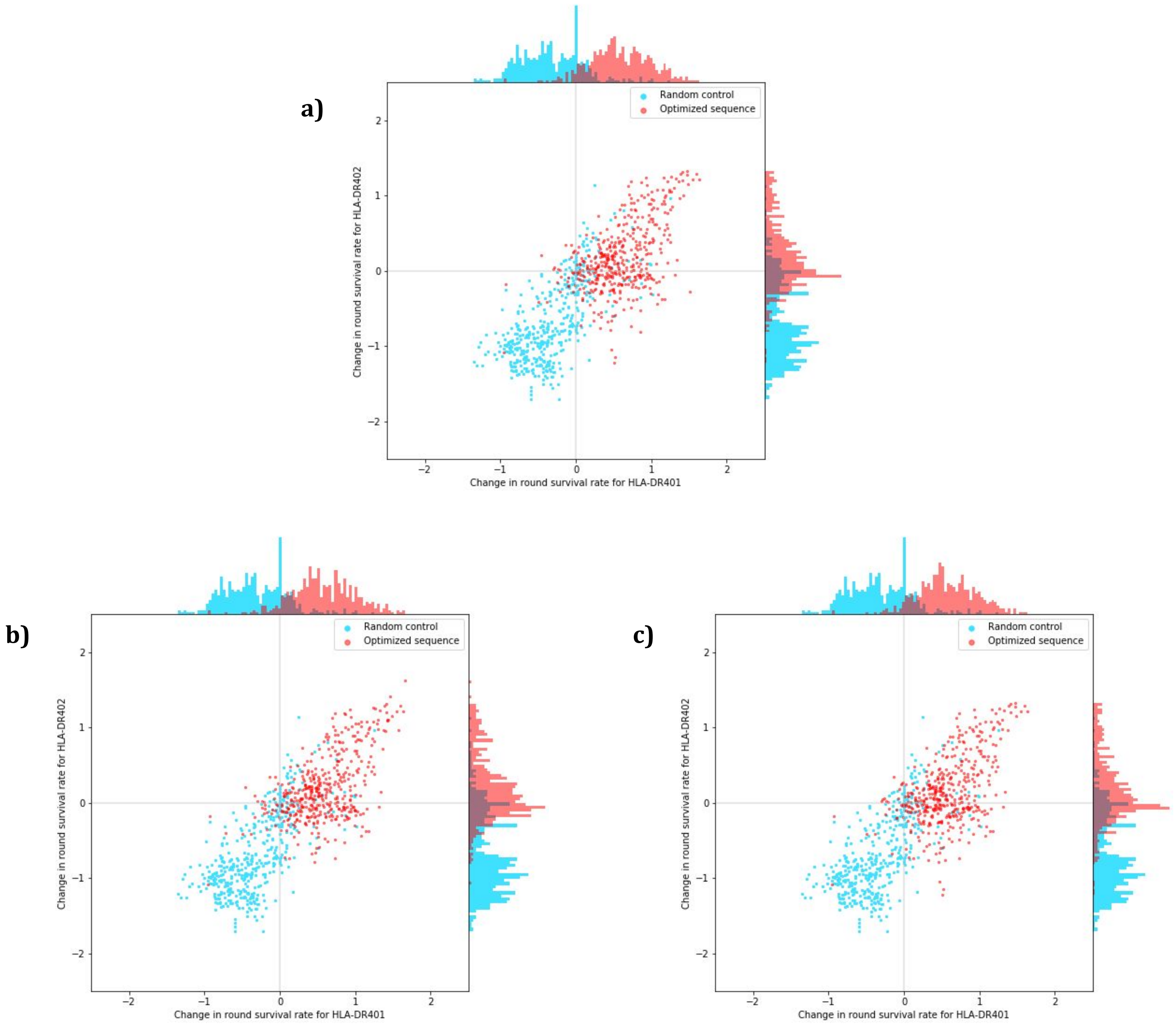

RSR changes in multiple allele optimization for other optimization methods. The same plots as Fig 4D, but for different optimization schemes. **a)** PE under the categorical model. **b)** UCB under the Gaussian model. **c)** UCB under the categorical model.

Supplemental Figure 5

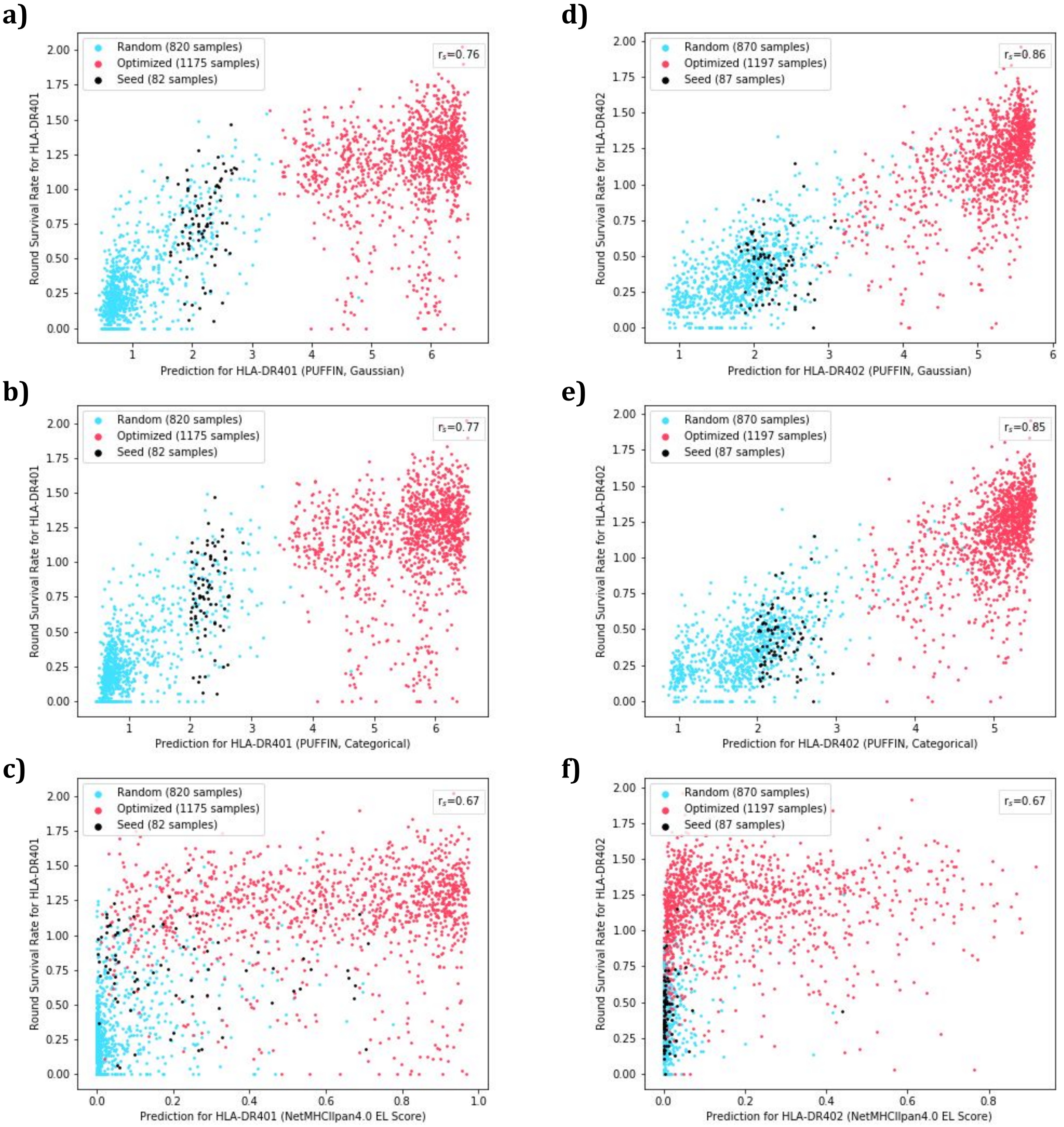

PUFFIN enrichment predictions correlate strongly with round survival rate. The measured RSR of seed, optimized, and randomly perturbed peptides are plotted against the outputs obtained from various predictors. The peptide's RSR for DR401 is plotted on the y-axis of (**a-c**), while the peptide's RSR for DR402 is plotted on the y-axis of (**d-f**). On the x-axis are the predicted values obtained from the Gaussian PUFFIN model (**a, d**) and the Categorical PUFFIN model (**b, e**), and the EL score from NetMHCPanII4.0 (**c, f**). Given in each plot are the Spearman correlation coefficients r_s. For each predictor, a higher predicted value should be indicative of better binding.

Supplemental Figure 6

a)

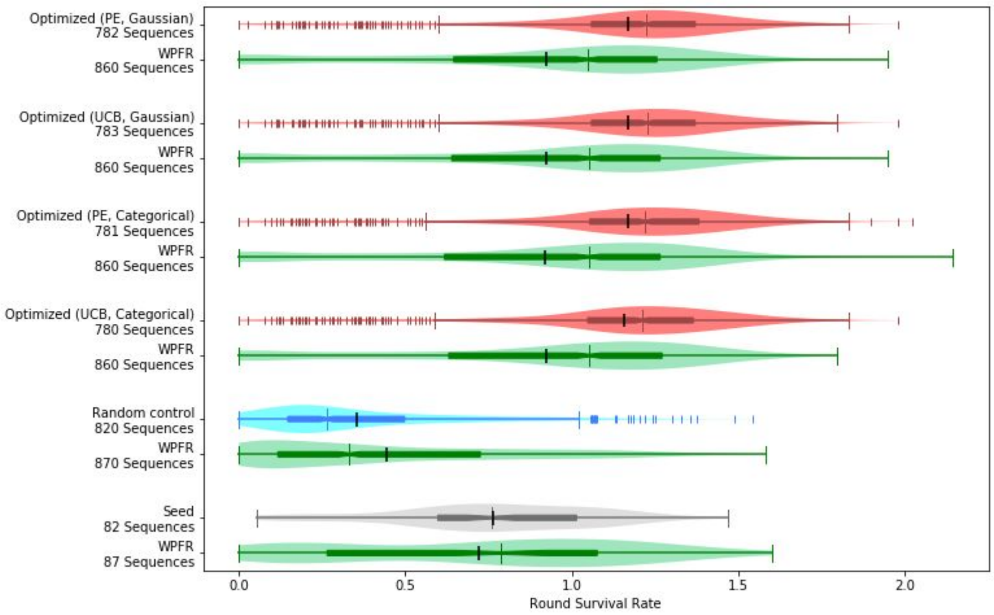

b)

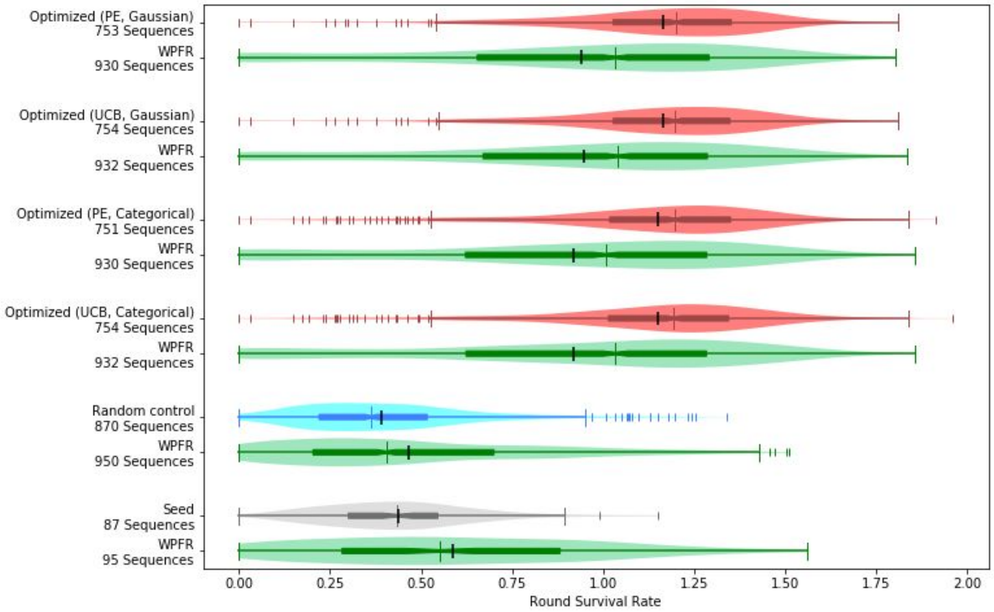

Comparing round survival rate of various groups with invariant peptide flanking residues and wild type peptide flanking residues. From top to bottom, the first four pairs of groups depicted in that figure are optimized sequences, and the pairs below them are sequences with random anchor mutations and seed sequences. The upper group of each pair contain IPFR flanked sequences and are the same as those in Figure 2, while the lower group in green contain WPFR flanked sequences. The distributions of RSR for **a)** HLA-DR401 and **b)** HLA-DR402 are plotted for these groups. Each plot is a combination of a box plot and a violin plot, where the distribution is shown by the violin plot in a lighter color, and the box plot shows the middle quartiles in a darker color along with the median. The mean is indicated by a black vertical line. Flier points are marked with the “|” symbol.

Supplemental Figure 7

a)

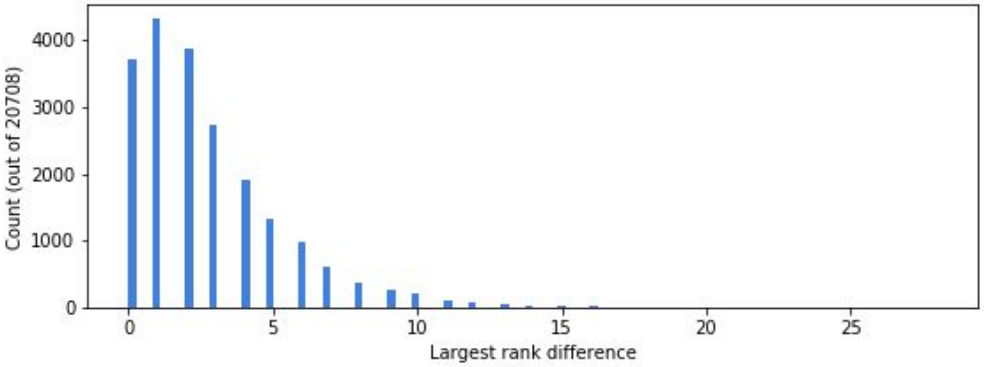

b)

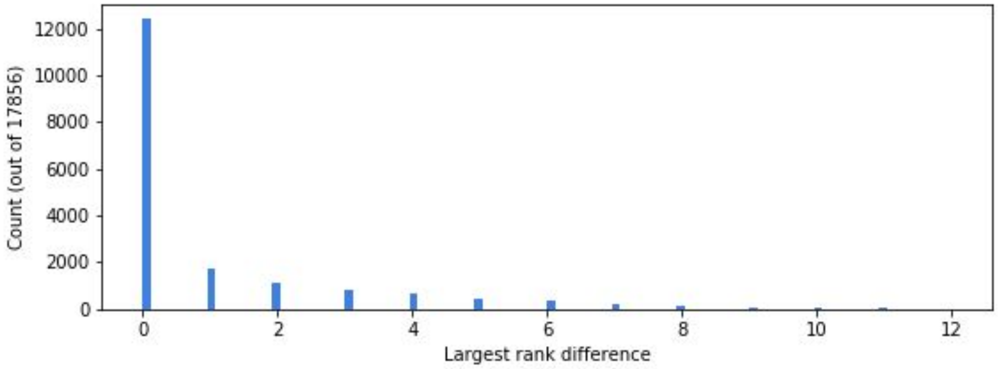

c)

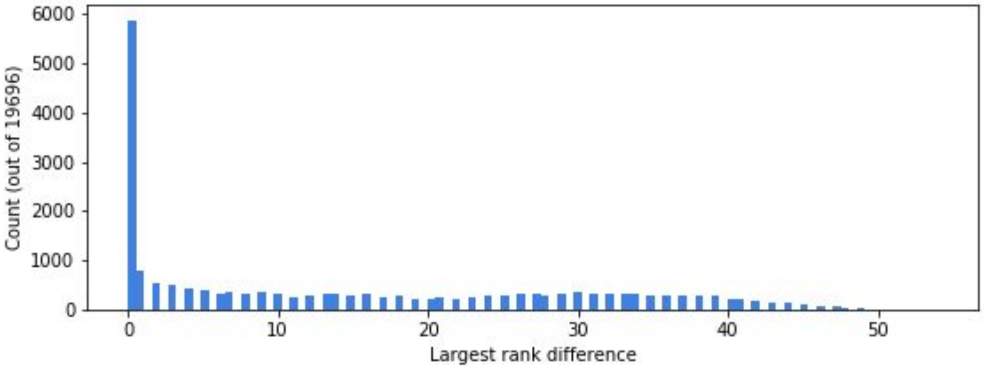

d)

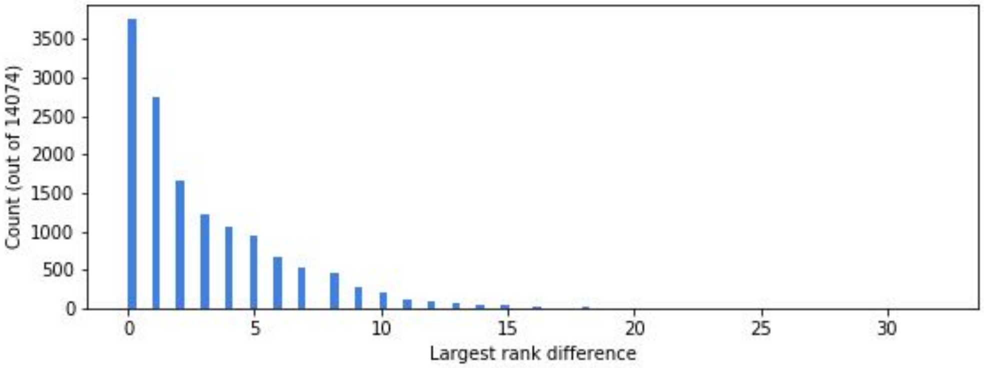

RSR convergence. We estimated RSR values for each set of data 23 times. For each run, we obtain an assignment from a read count vector to an RSR value. This gives us an ordering over the read count vectors, and allows us to assign a rank to each unique read count vector. For each read count vector, we compute the largest discrepancy in its rank between the 23 rounds. The histogram depicts the distribution of these discrepancies. **a)** Validation sequences for HLA-DR401. **b)** Validation sequences for HLA-DR402. **c)** Training sequences for HLA-DR401. **d)** Training sequences for HLA-DR402.

Supplemental Figure 8

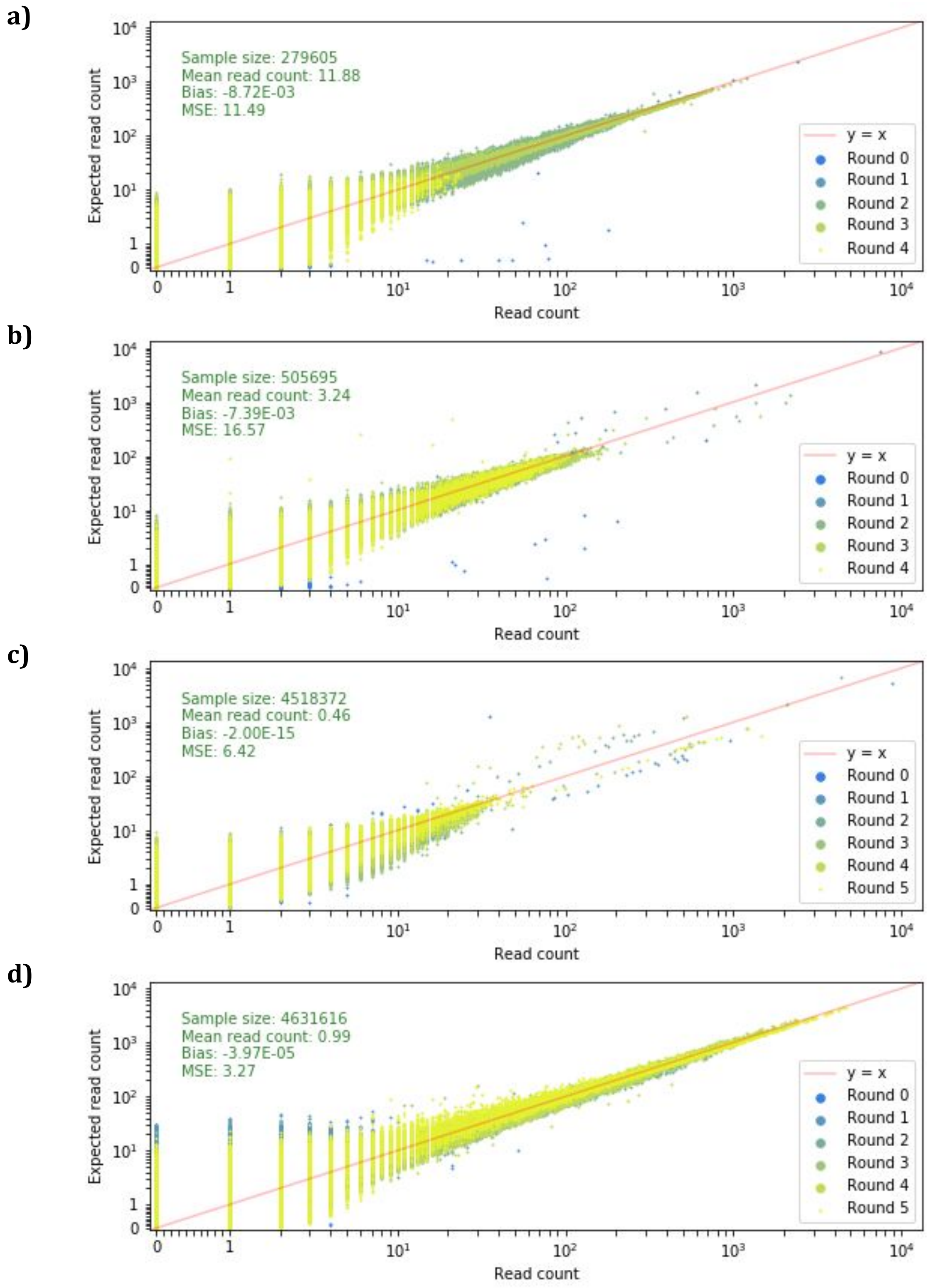

RSR model fit. Given the estimated parameters of our MAP estimate, we plot actual read count against the expected read count under the model. **a)** Validation sequences for HLA-DR401. **b)** Validation sequences for HLA-DR402. **c)** Training sequences for HLA-DR401. **d)** Training sequences for HLA-DR402.

Supplemental Figure 9

a)

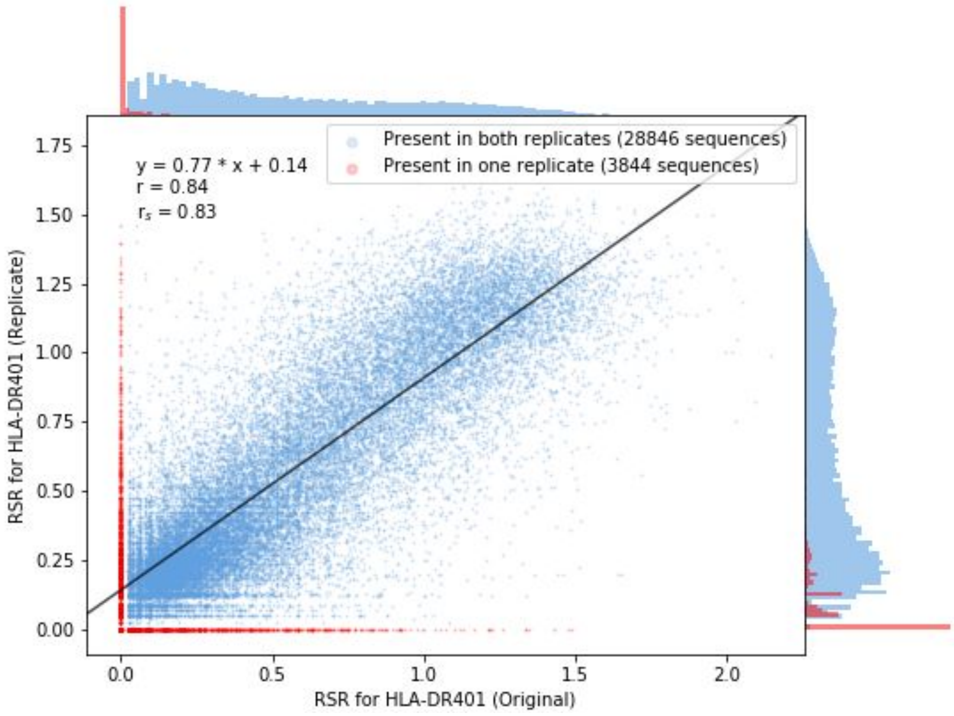

b)

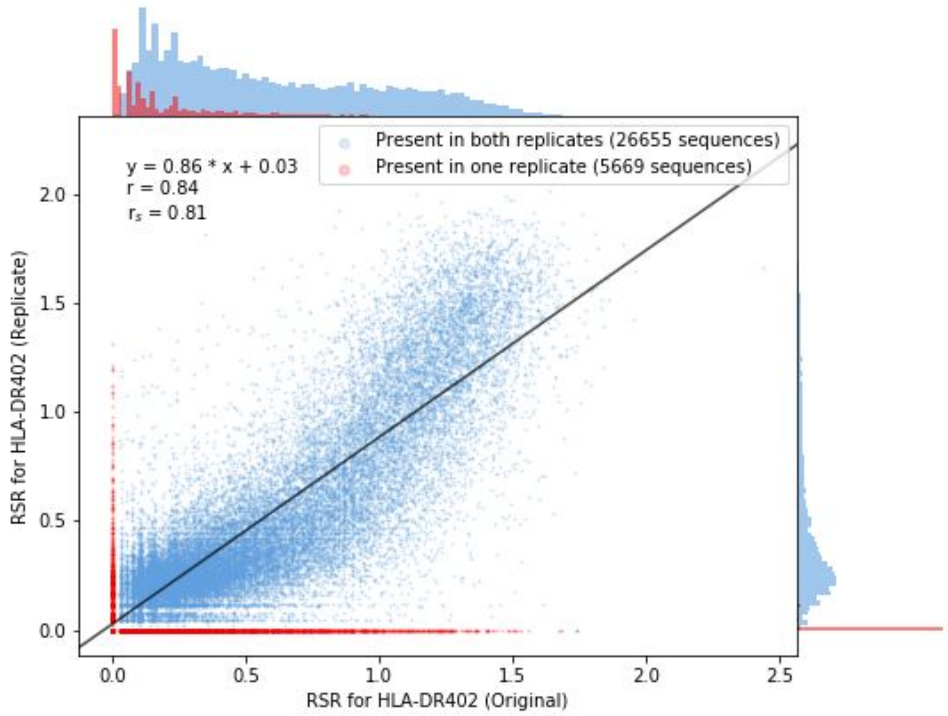

Validation through replicates. We validated the RSR values from the validation round by performing a second replicate. **a)** We plot the RSR values between the original and replicate rounds for HLA-DR401. If a point is not present in one of the experiments, it is given a value of 0 and marked in red. The line of best fit obtained from linear regression for points that were present in both experiments, and is shown alongside the Pearson correlation coefficient r and the Spearman correlation coefficient r_s. **b)** We plot the RSR values between the original and replicate rounds for HLA-DR402.

Supplemental Figure 10

Optimized Set	PE, Gaussian	UCB, Gaussian	PE, Categorical	UCB, Categorical	NetMHCII pan4.0	Random Neighbor
HLA-DR401 PE, Gaussian	82/82	82/82	82/82	82/82	57/82	0/82
HLA-DR401 UCB, Gaussian	82/82	82/82	82/82	82/82	50/82	0/82
HLA-DR401 PE, Categorical	82/82	82/82	82/82	80/82	65/82	0/82
HLA-DR401 UCB, Categorical	82/82	82/82	80/82	82/82	57/82	0/82
HLA-DR401 NetMHCIIpanII4.0	57/82	50/82	65/82	57/82	82/82	0/82
HLA-DR401 Random Control	0/82	0/82	0/82	0/82	0/82	82/82
HLA-DR402 PE, Gaussian	87/87	87/87	86/87	85/87	45/87	0/87
HLA-DR402 UCB, Gaussian	87/87	87/87	84/87	85/87	44/87	0/87
HLA-DR402 PE, Categorical	86/87	84/87	87/87	87/87	52/87	0/87
HLA-DR402 UCB, Categorical	85/87	85/87	87/87	87/87	49/87	0/87
HLA-DR402 NetMHCIIpan4.0	45/87	44/87	52/87	49/87	87/87	0/87
HLA-DR402 Random Control	0/87	0/87	0/87	0/87	0/87	87/87
Joint PE, Gaussian	44/44	44/44	44/44	44/44	27/44	0/44
Joint UCB, Gaussian	44/44	44/44	44/44	44/44	25/44	0/44
Joint PE, Categorical	44/44	44/44	44/44	44/44	27/44	0/44
Joint UCB, Categorical	44/44	44/44	44/44	44/44	26/44	0/44
Joint NetMHCIIpan4.0	27/44	25/44	27/44	26/44	44/44	0/44
Joint Random Control	0/44	0/44	0/44	0/44	0/44	44/44

Overlapping predictions from alternate prediction algorithms. Each column indicates a proposed optimization method, and each row indicates a proposed optimization. Each entry indicates the number of seeds with overlapping proposed peptides between optimization methods (out of 82, 87, and 44 seed sequences for HLA-DR401, HLA-DR402, and joint optimization, respectively). "PE, Gaussian", "UCB, Gaussian", "PE, Categorical", and "UCB, Categorical" are the main optimization methods we analyzed which use PUFFIN predictions, NetMHCIIpan4.0 uses the EL score predicted by NetMHCIIpan as an objective function but otherwise operates identically to the other optimization methods, and "Random Control" draws anchor substitutions randomly.
